# Jaw claudication and branch perfusion reduction as rare complications of fenestrated thoracic endovascular aortic repair

**DOI:** 10.1016/j.jvscit.2024.101484

**Published:** 2024-03-14

**Authors:** Ryota Nakano, Shinichi Iwakoshi, Sho Shimizu, Takahiro Nakai, Shigeo Ichihashi, Toshihiro Tanaka

**Affiliations:** Department of Diagnostic and Interventional Radiology, Nara Medical University, Kashihara, Japan

**Keywords:** Aortic arch aneurysms, Brachiocephalic artery, Fenestrated thoracic endovascular aortic repair, Graft protrusion, Jaw claudication

## Abstract

We report a rare case of jaw claudication following fenestrated thoracic endovascular aortic repair for a saccular aortic arch aneurysm. The brachiocephalic artery (BCA) was preserved with fenestration and intentionally half covered. Although discharged without any complications 2 weeks after the procedure, the patient subsequently experienced right mandibular fatigue at mealtime and hypotension in the right upper extremity. Angiography revealed a flap-like structure in the BCA orifice, with a 100-mm Hg pressure gradient between the aorta and BCA. Intravascular ultrasound revealed a stenosed BCA with a cord-like structure, which was considered a graft protrusion. Bare metal stenting was performed, which promptly resolved the symptoms.

Open surgery is considered the gold standard for the treatment of aortic arch aneurysms. However, depending on the patient's condition and comorbidities, thoracic endovascular aortic repair (TEVAR) could be an option. TEVAR for aortic arch aneurysms requires preservation of supra-aortic branch blood flow, and fenestrated TEVAR is one solution. Common fenestrated TEVAR complications include stroke, endoleaks, access site complications, guidewire injuries, retrograde dissections, renal injury, unintentional great vessel coverage, spinal cord ischemia, and device failure.^1^ Brachiocephalic artery (BCA) stenosis is a rare complication after fenestrated TEVAR and is mainly caused by device migration, device failure, fenestration mismatch, or unintentional great vessel coverage. Common BCA stenosis symptoms are ischemic symptoms in its branches, including ataxia, dizziness, upper extremity claudication, syncope, transient ischemic attack, vertigo, and visual disturbances.^2^ To the best of our knowledge, jaw claudication has not been reported previously as a complication of fenestrated TEVAR. Hence, in this case report, we report jaw claudication as a rare complication following fenestrated TEVAR that was caused by graft protrusion into the BCA.

## Case report

**Fig 1:**
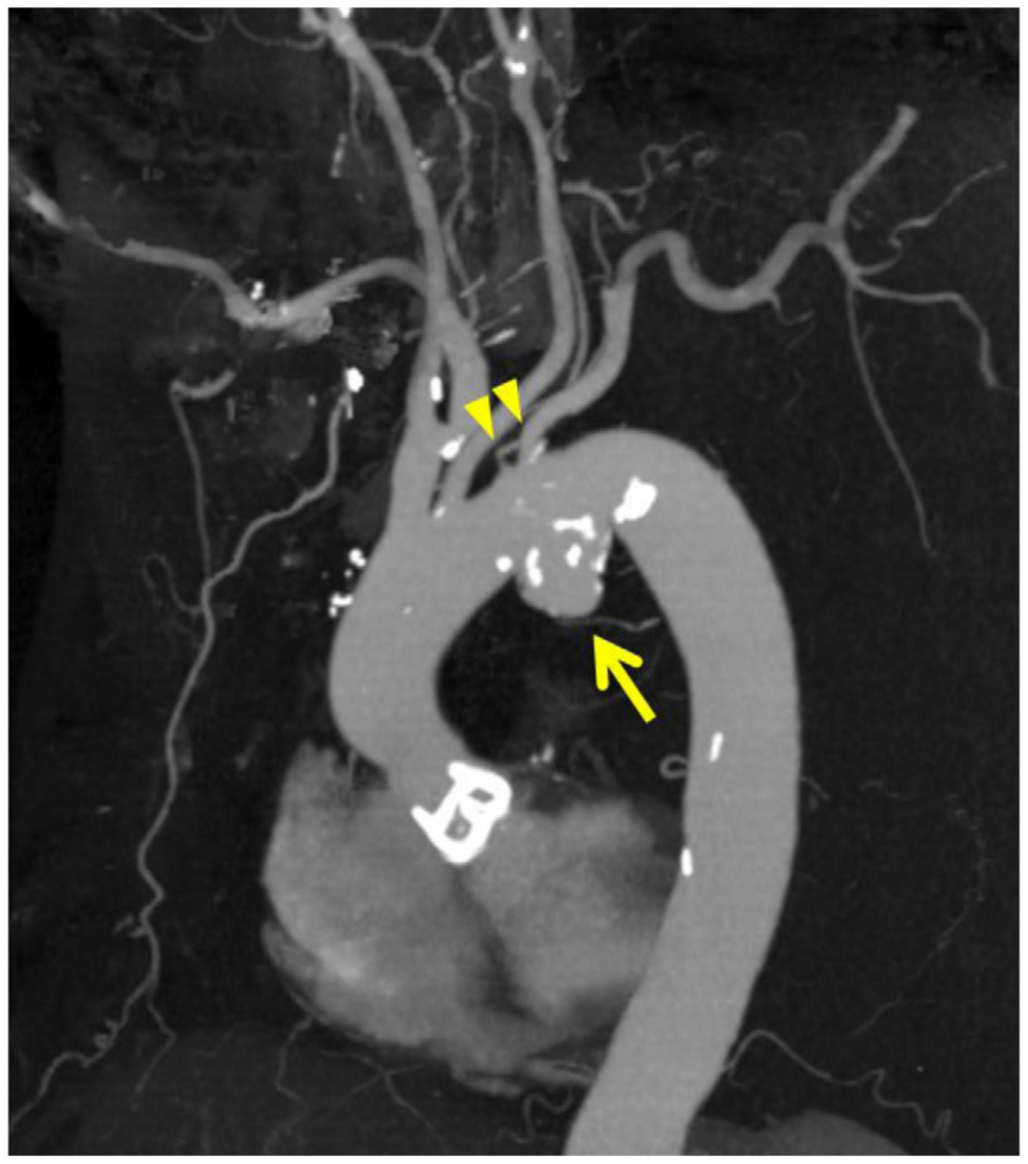
Three-dimensional reconstruction of the thoracic aorta from computed tomography (CT) angiography before thoracic endovascular aortic repair (TEVAR). CT angiography showed a saccular aneurysm of the aortic arch (*arrow*). The maximum diameter of the aneurysm was 52 mm. The left vertebral artery branched directly from the aorta. The left subclavian artery and left vertebral artery (*arrowheads*) branched at the same level as the aortic aneurysm.

A 77-year-old man with hypertension was referred to our hospital for thoracic aortic aneurysm treatment. Contrast-enhanced computed tomography (CT) revealed a saccular aneurysm of the aortic arch measuring 52 mm in maximum diameter. The left vertebral artery branched directly from the aorta, and the left subclavian artery and left vertebral artery branched at the same level as the aortic aneurysm (Fig 1). After careful discussion, it was decided to perform TEVAR using a Najuta stent graft (Kawasumi Laboratories, Inc) because of the advanced age of the patient and a surgical history for severe aortic valve stenosis. The patient provided written informed consent for the procedure and the report of his case details and imaging studies, including anatomic information on the proximal and distal landing zones.

**Fig 2:**
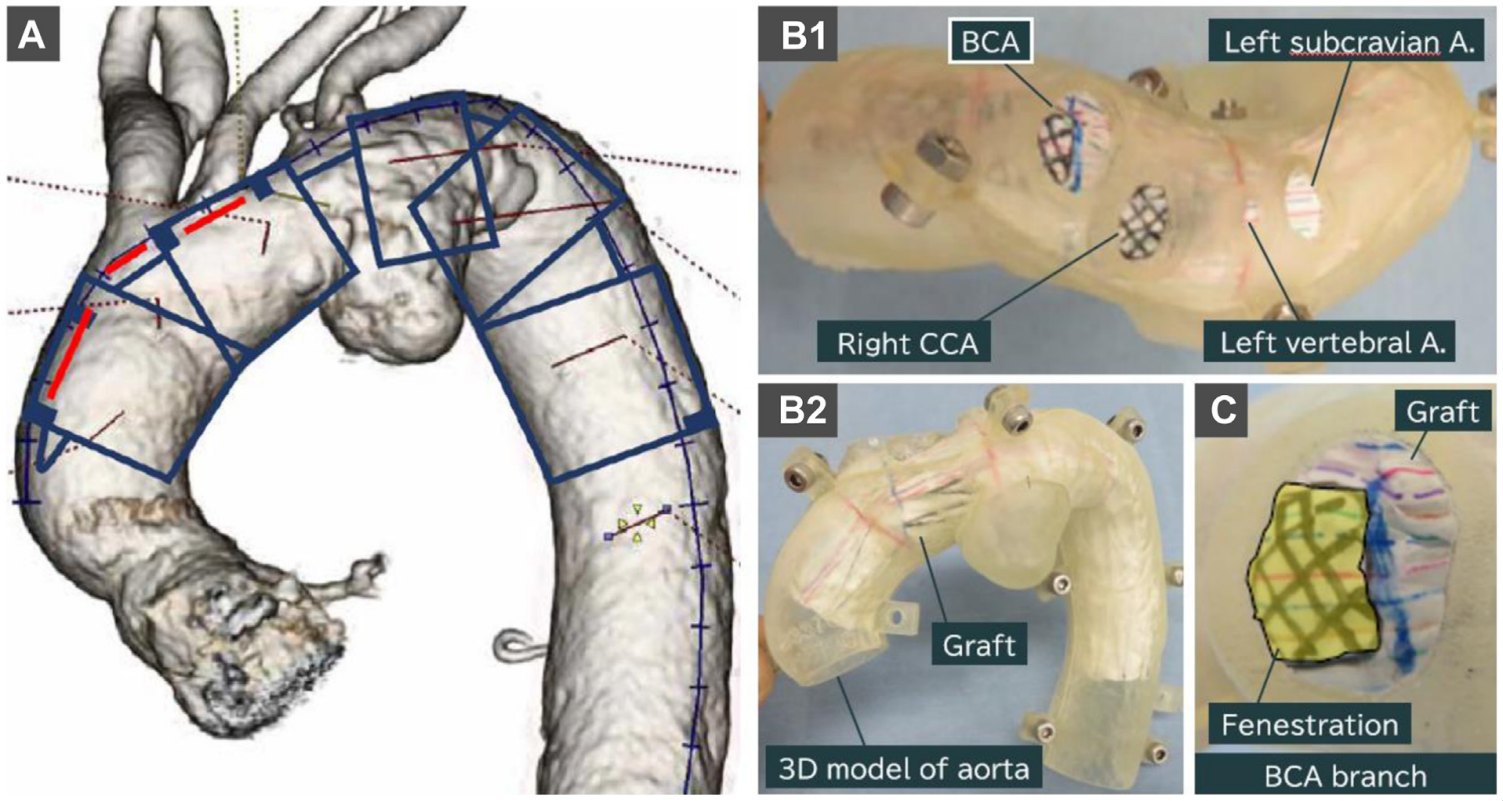
Preoperative plan images. **A,** Imaging of the deployment position of the Najuta stent graft. *Red lines* demonstrate fenestration positions. The left subclavian artery and left vertebral artery are close to the aneurysm; therefore, these two branches are planned to be intentionally obstructed. **B,** Simulation image of the stent graft deployment into the three-dimensional model of the aorta from top (**B1**) and front (**B2**) views. The *top view* shows the orifice of each aortic branch. The *shaded areas* indicate fenestrations, which are planned to be located at the brachiocephalic artery (BCA) and right common carotid artery (CCA) orifice. **C,** Cross section of the BCA branch. *Yellow* indicates the area to be preserved by the fenestration. Note the brachiocephalic orifice is planned to be half covered. *A.,* Artery.

In the preoperative plan, a Najuta stent graft with two fenestrations was prepared to preserve the BCA and left common carotid artery (CCA) perfusion. Because of its proximity to the left CCA, it was planned for the BCA to be half covered with a graft (Fig 2).

**Fig 3:**
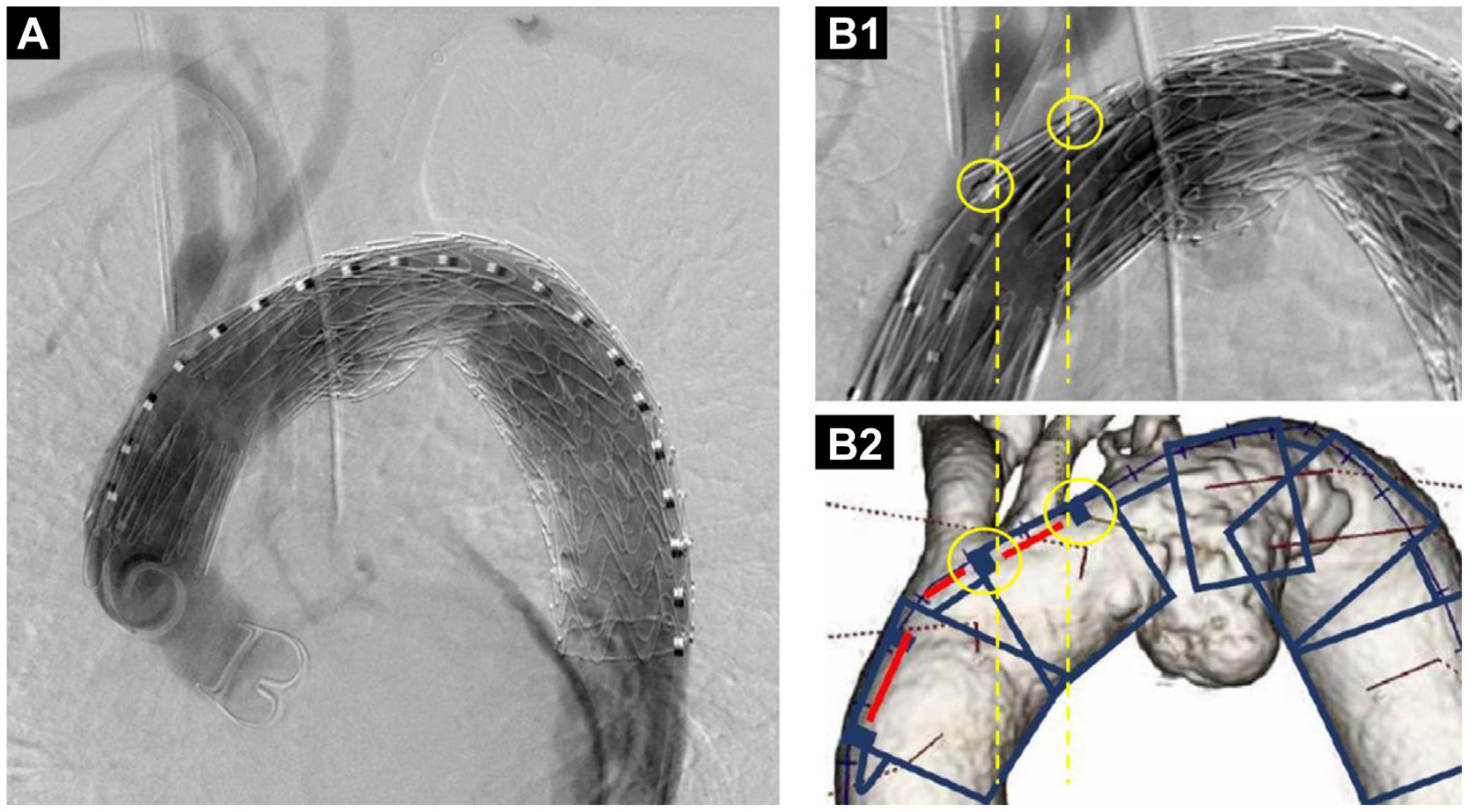
Aortographic images of thoracic endovascular aortic repair (TEVAR) with a Najuta stent graft. **A,** Aortography after deploying the Najuta stent graft showing no delay or reduction of contrast medium in the brachiocephalic artery (BCA). **B,** Image showing deployment of the Najuta stent graft (**B1**) compared with the preoperative image (**B2**). Markers of second stent of the Najuta graft matched the location of the preoperative plan (*yellow circles*).

Under general anesthesia, cardiovascular surgeons bypassed the left CCA and left axillary artery. The distal part of the left subclavian artery was punctured, and a 6F guiding sheath (45-cm Destination; Terumo Corp) was inserted. The right brachial artery was percutaneously punctured under ultrasound guidance, and a 4.5F guiding sheath (Parent Plus 30; Medikit) was inserted. The left common femoral artery (CFA) was exposed and punctured, and a 20F DrySeal sheath (W.L. Gore & Associates) was inserted. A 20 × 15-cm cTAG (W.L. Gore & Associates) was deployed slightly distal to the left CCA. The 20F DrySeal sheath was replaced with a 24F DrySeal sheath. A through-and-through wire was established from the left CFA to the right brachial artery using a 0.035-in. (0.09-cm) guidewire. The Najuta stent graft was advanced and deployed from the ascending aorta per the preoperative plan (Fig 3). To prevent a type II endoleak, the orifice of the left subclavian artery was embolized using detachable coils (Interlock coils, 12 mm, 8 mm; Boston Scientific). Completion angiography revealed no endoleak and good perfusion of the BCA and left CCA without any delay.

The patient was hospitalized for 2 weeks postoperatively and discharged after the postoperative inflammatory response had improved. There were no complications during hospitalization. At a follow-up examination 4 weeks after discharge, the patient complained of right upper extremity claudication, in addition to right mandibular fatigue at mealtime. A difference in blood pressure between the upper arms was detected, which had not been observed preoperatively. Consequently, malperfusion of the BCA was suspected and was also considered as a cause of these symptoms. Contrast-enhanced CT revealed no Najuta stent graft migration. Angiography was performed to determine the cause of the hypoperfusion. In addition, simultaneous pressure measurement between the BCA and aortic arch was planned.

**Fig 4:**
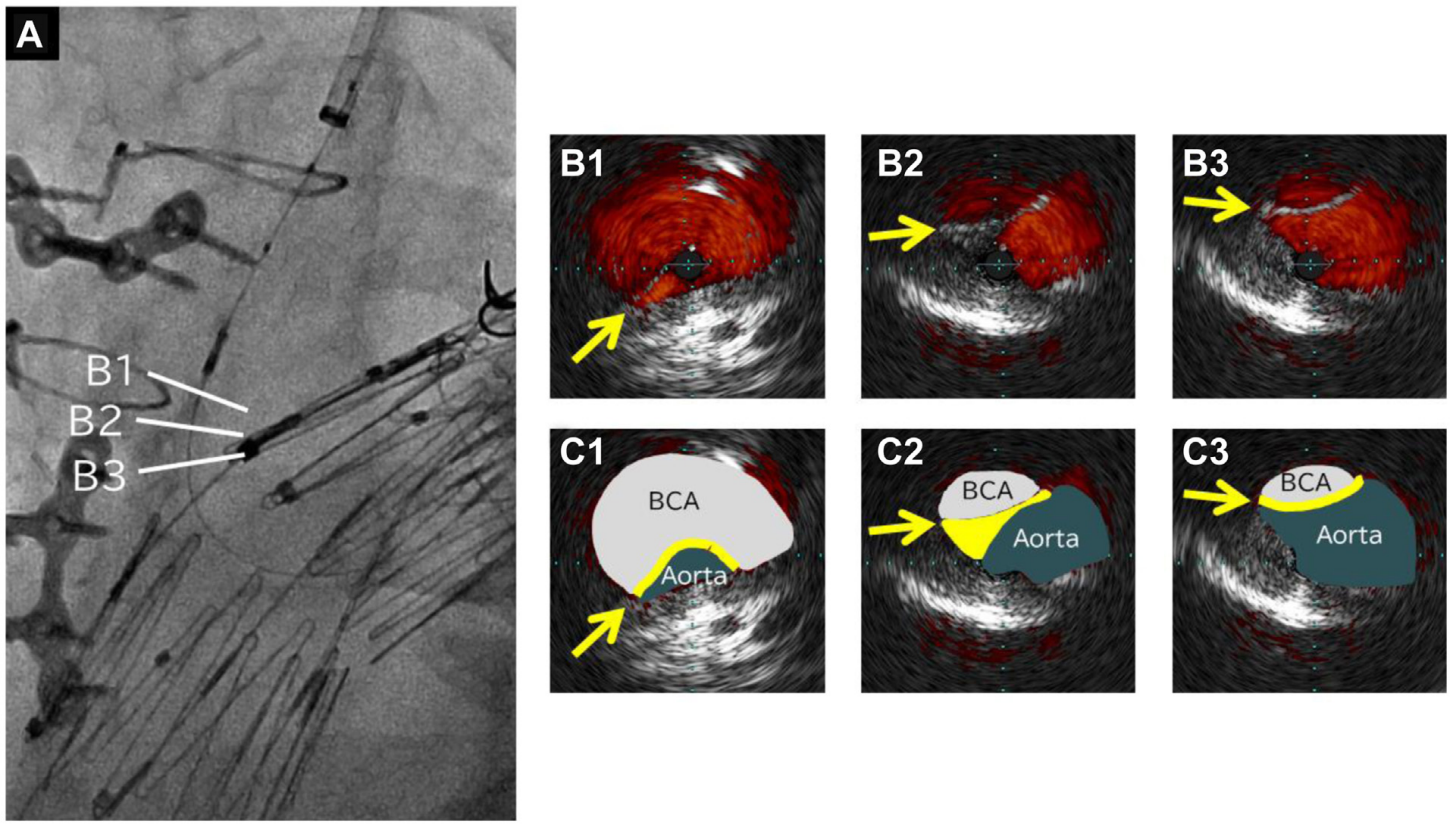
**A,** Angiography of the intravascular ultrasound catheter. **B,** Intravascular ultrasound images arranged in sequence from the brachiocephalic artery (BCA) toward the aorta: **B1-B3** and **C1-C3** show the cord-like structure stenosing the BCA (*arrow*).

**Fig 5:**
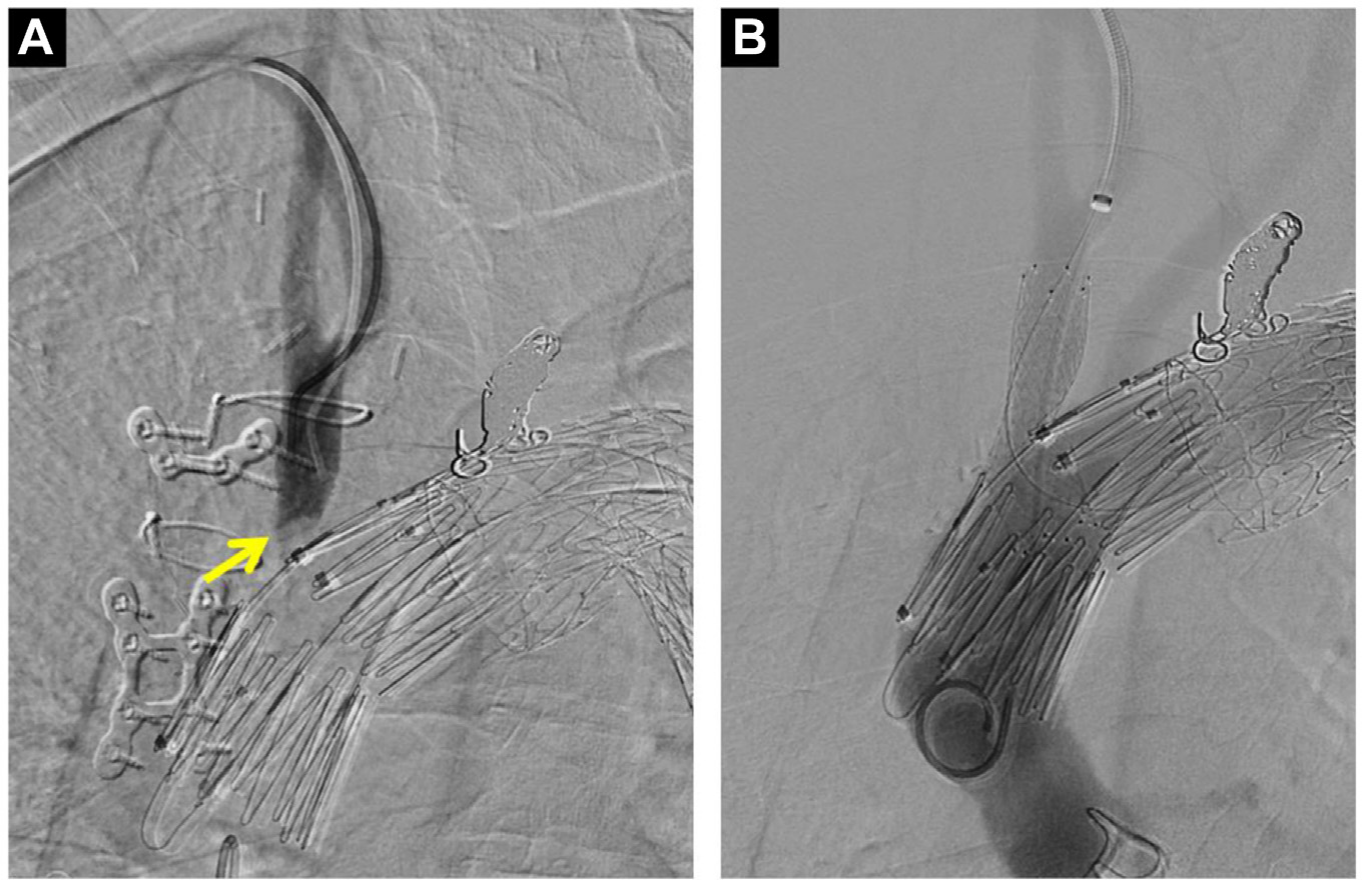
Angiographic images before and after brachiocephalic artery (BCA) stenting. **A,** Angiographic image with contrast medium introduced into the BCA showing an arc-shaped contrast defect. It was considered that the membranous structure prevented blood flow from the aorta. **B,** Aortography after BCA stenting showing no delay or reduced contrast in the BCA.

The right CFA and right brachial artery were percutaneously punctured under ultrasound guidance, and a 4F sheath was inserted into each artery. The pressure gradient between the aortic arch and the BCA was 100 mm Hg. No stent graft migration was seen on angiography compared with the perioperative images. BCA angiography demonstrated an arc-shaped contrast defect at the BCA orifice. The 4F sheath at the right brachial artery was changed to a 7F Flexor Ansel sheath (Cook Medical). A through-and-through wire was established between the right CFA and right brachial artery using a 0.014-in. (1.56-cm) guidewire. Intravascular ultrasound revealed that the BCA was stenosed with a cord-like structure. We considered that the hypotension of his right upper limb was caused by graft protrusion into the BCA (Fig 4). We deployed a bare nitinol stent (S.M.A.R.T., 12 mm, 4 cm; Cordis) into the BCA orifice, successfully pushing the covering graft aside and opening the BCA (Fig 5). The aorta and BCA pressure gradient disappeared. The symptoms resolved promptly after stenting and had not worsened at 1 year after stenting.

## Discussion

In the present case, angiography during the first endovascular treatment revealed that the Najuta stent graft was placed according to the preoperative plan, with well-preserved BCA blood flow. Subsequent angiography demonstrated no migration of the Najuta stent graft; however, an arc-shaped contrast defect was seen at the BCA orifice, which was recognized as a cord-like structure on the intravascular ultrasound image. Based on these findings, the cord-like structure was identified as the prosthesis graft, which expanded toward the BCA because of aortic pressure. The stent and graft of the Najuta stent graft are sutured only at the proximal and distal ends,^3^ allowing the prosthesis graft to expand toward the aortic wall, contributing to better sealing. However, the expanded graft protruded into the BCA in this case, likely causing decreased BCA perfusion. It is important to note that when half covering the branch arteries with these types of stent grafts, attention should be given to the perfusion of these arteries, as this could cause stenosis due to graft protrusion into these arteries.

**Fig 6:**
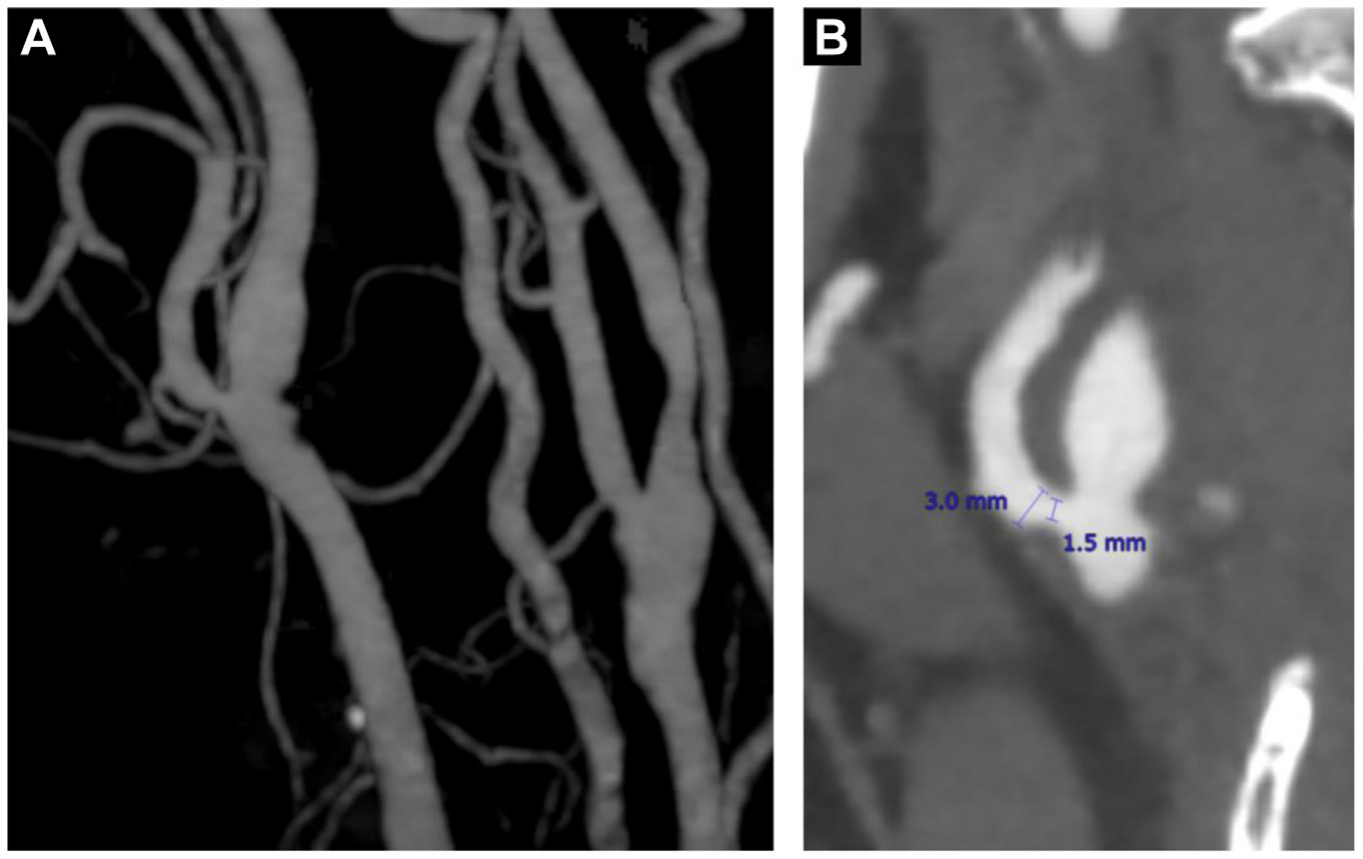
**A,** Three-dimensional reconstruction of the right external carotid artery (ECA) from computed tomography (CT) angiography before thoracic endovascular aortic repair (TEVAR). **B,** CT angiography showing right ECA stenosis.

In this case, jaw claudication occurred after the fenestrated TEVAR and promptly resolved after BCA stenting. Therefore, the jaw claudication was likely caused by BCA stenosis. Jaw claudication is a well-known symptom of giant cell arteritis and is also known as the symptom of occlusive external carotid artery (ECA) disease.^4^ Carotid artery stenting can sometimes cause ECA stenosis or occlusion,^5^ which can cause jaw claudication after carotid artery stenting.^6^^,^^7^ Jaw claudication after TEVAR has not been reported, and jaw claudication caused by BCA stenosis is uncommon. Jaw claudication is considered to be caused by ischemia from decreased blood flow through the facial ECA branches.^8^ Because the ECA is a branch of the BCA, BCA stenosis can reduce ECA blood flow and cause ischemic ECA symptoms, including jaw claudication. In this case, preoperative CT angiography revealed mild stenosis of the right ECA (Fig 6). Stenosis of the BCA and right ECA likely accounted for the jaw claudication. If jaw claudication occurs after treating major vascular or carotid artery issues, evaluating blood flow disturbances from the BCA to the ECA would be beneficial.

## Conclusions

Jaw claudication was caused by BCA stenosis following fenestrated TEVAR for an aortic arch aneurysm. Physicians should be aware that partial coverage of branch arteries during the procedure could potentially reduce perfusion because of graft protrusion, and jaw claudication is a possible complication after fenestrated TEVAR.

## Disclosures

None.
